# Kinetics and Persistence of the Cellular and Humoral Immune Responses to BNT162b2 mRNA Vaccine in SARS-CoV-2-Naive and -Experienced Subjects: Impact of Booster Dose and Breakthrough Infections

**DOI:** 10.3389/fimmu.2022.863554

**Published:** 2022-05-31

**Authors:** Salomé Desmecht, Aleksandr Tashkeev, Majdouline El Moussaoui, Nicole Marechal, Hélène Perée, Yumie Tokunaga, Celine Fombellida-Lopez, Barbara Polese, Céline Legrand, Marie Wéry, Myriam Mni, Nicolas Fouillien, Françoise Toussaint, Laurent Gillet, Fabrice Bureau, Laurence Lutteri, Marie-Pierre Hayette, Michel Moutschen, Christelle Meuris, Pieter Vermeersch, Daniel Desmecht, Souad Rahmouni, Gilles Darcis

**Affiliations:** ^1^Laboratory of Animal Genomics, Grappe Interdisciplinaire de Génoprotéomique Appliquée-Institute (GIGA)-Medical Genomics, Grappe Interdisciplinaire de Génoprotéomique Appliquée-Institute (GIGA)-Institute, University of Liège, Liège, Belgium; ^2^ Laboratory of Infectious Diseases, Grappe Interdisciplinaire de Génoprotéomique Appliquée-Institute (GIGA)-I3, Grappe Interdisciplinaire de Génoprotéomique Appliquée-Institute (GIGA)-Institute, University of Liège, Liège, Belgium; ^3^Department of Infectious Diseases, Liège University Hospital, Liège, Belgium; ^4^Laboratory of Cellular and Molecular Immunology, Grappe Interdisciplinaire de Génoprotéomique Appliquée-Institute (GIGA)-I3, Grappe Interdisciplinaire de Génoprotéomique Appliquée-Institute (GIGA) Institute, University of Liège, Liège, Belgium; ^5^Department of Clinical Microbiology, University Hospital of Liège, Liège, Belgium; ^6^ Immunology-Vaccinology Laboratory, Department of Infectious and Parasitic Diseases, Fundamental and Applied Research for Animals & Health (FARAH), University of Liège, Liège, Belgium; ^7^Clinical Chemistry Department, University Hospital of Liège, Liège, Belgium; ^8^Clinical Department of Laboratory Medicine and National Reference Center for Respiratory Pathogens, University Hospitals Leuven, Leuven, Belgium; ^9^Department of Animal Pathology, Fundamental and Applied Research for Animals & Health (FARAH), Liège University, Liège, Belgium

**Keywords:** COVID-19, BNT162b2 mRNA vaccine, IFN-γ, neutralizing antibodies, SARS- CoV-2

## Abstract

**Background:**

Understanding and measuring the individual level of immune protection and its persistence at both humoral and cellular levels after SARS-CoV-2 vaccination is mandatory for the management of the vaccination booster campaign. Our prospective study was designed to assess the immunogenicity of the BNT162b2 mRNA vaccine in triggering the cellular and humoral immune response in healthcare workers up to 12 months after the initial vaccination, with one additional boosting dose between 6 and 12 months.

**Methods:**

This prospective study enrolled 208 healthcare workers (HCWs) from the Liège University Hospital (CHU) of Liège in Belgium. Participants received two doses of BioNTech/Pfizer COVID-19 vaccine (BNT162b2) and a booster dose 6-12 months later. Fifty participants were SARS-CoV-2 experienced and 158 were naïve before the vaccination. Blood sampling was performed at the day of the first (T0) and second (T1) vaccine doses administration, then at 2 weeks (T2), 4 weeks (T3), 6 months (T4) and 12 months (T5) after the second dose. Between T4 and T5, participants also got the third boosting vaccine dose. A total of 1145 blood samples were collected. All samples were tested for the presence of anti-Spike antibodies, using the DiaSorin LIAISON SARS-CoV-2 Trimeric S IgG assay, and for anti-Nucleocapsid antibodies, using Elecsys anti-SARS-CoV-2 assay​​. Neutralizing antibodies against the SARS-CoV-2 Wuhan-like variant strain were quantified in all samples using a Vero E6 cell-based neutralization assay. Cell-mediated immune response was evaluated at T4 and T5 on 80 and 55 participants, respectively, by measuring the secretion of IFN-γ on peripheral blood lymphocytes using the QuantiFERON Human IFN-γ SARS-CoV-2, from Qiagen. We analyzed separately the naïve and experienced participants.

**Findings:**

We found that anti-spike antibodies and neutralization capacity levels were significantly higher in SARS-CoV-2 experienced HCWs compared to naïve HCWs at all time points analyzed except the one after boosting dose. Cellular immune response was also higher in experienced HCWs six months following vaccination. Besides the impact of SARS-CoV-2 infection history on immune response to BNT162b2 mRNA vaccine, we observed a significant negative association between age and persistence of humoral response. The booster dose induced an increase in humoral and cellular immune responses, particularly in naive individuals. Breakthrough infections resulted in higher cellular and humoral responses after the booster dose.

**Conclusions:**

Our data strengthen previous findings demonstrating that immunization through vaccination combined with natural infection is better than 2 vaccine doses immunization or natural infection alone. The benefit of the booster dose was greater in naive individuals. It may have implications for personalizing mRNA vaccination regimens used to prevent severe COVID-19 and reduce the impact of the pandemic on the healthcare system. More specifically, it may help prioritizing vaccination, including for the deployment of booster doses.

## Introduction

Mass vaccination of the population plays a crucial role in the control of the coronavirus disease 2019 (COVID-19) pandemic. Pioneering studies have shown that the BNT162b2 (Pfizer–BioNTech), as well as the mRNA-1273 (Moderna) vaccines provide strong protective efficacy against COVID-19 in subjects of 5 years old and older and are highly effective in the first few months after vaccination against documented infection and symptomatic COVID-19 (1–6). Nevertheless, several studies indicate that immunity gradually waned in all age groups a few months after having received the second dose of vaccine (7–10). Indeed, 8 months after COVID-19 mRNA vaccination, the median live-virus neutralizing antibody titer, pseudovirus neutralizing antibody titer, and RBD-specific binding antibody titer elicited by the vaccine are significantly lower than the peak titers (11). As a consequence, the rate of confirmed infection among persons vaccinated revealed a substantial increase as the time from vaccination increased (7–10). Though, COVID-19 mRNA vaccines-induced protection against hospitalization and death persisted with barely any waning for 6 months after the second dose (7–10), suggesting that persisting cellular immunity drives the immune response and prevents viral dissemination when antibodies wane. T cell responses persist up to 6 months after vaccination, with the maintenance of a pool of polyfunctional memory antigen-specific T cells (11–13). mRNA vaccines also produce persisting functional memory B cells (13).

Although the efficacy of the vaccine against severe disease, hospitalization, and death remains high, weakening immunity and emergence of variants of concern create a need for a third vaccine dose (14). Indeed, a recent study demonstrated that such booster vaccination induces neutralizing immunity even against the new SARS-CoV-2 Omicron harboring 34 mutations more than all the other variants (15). Additional studies have demonstrated a rapid and consistent reduction in the COVID-19 burden among persons living in long-term care facilities after the initiation of a BNT162b2 booster campaign (16) as well as in other groups of age (17). However, information on how pre-existing immunity to SARS-CoV-2 would be boosted by mRNA vaccination remains poorly understood. In particular, the helpfulness and the timing of booster vaccine doses remain to be determined as well as the impact of recovery after SARS-CoV-2 infection. Here, we analyzed the kinetic of humoral response after BNT162b2 vaccination and booster dose. We also analyzed cellular response to BNT162b2 up to 12 months after the vaccination and after booster dose. In particular, we studied the impact of previous and breakthrough SARS-CoV-2 infection on response to vaccination, including booster dose. *De novo* SARS-CoV-2 infection was monitored in all participants by measuring anti-Nucleocapsid antibodies at all time points analyzed.

## Methods

### Participant Enrollment and Blood Sampling

Adults (>18 years old) consenting hospital staff members (including health care workers and administrative staff) of CHU of Liège were invited to participate in the study. Participants were enrolled during February 2021. All participants received two doses of 0.3 mL of BNT162b2 mRNA vaccine administered to the deltoid muscle with a recommended dose interval of three to four weeks (IQR = 21-22 days), and then a third boosting dose a year after the first one (IQR = 346-352 days) (**Figure S1**). Demographics and clinical data were collected through a questionnaire. Participants were classified into two groups: “experienced group”, that includes participants with confirmed SARS-CoV-2 infection, and “naïve group”, consisting of individuals without previous SARS-CoV-2 infection. Previous infection was documented with the anti-Nucleocapsid IgG test from Roche (Elecsys Anti-SARS-CoV-2) (**Figure S2**). Fourteen participants were infected between T4 and T5 while only one participant was infected between T3 and T4.

Samples were treated accordingly as “naive” before infection, and “experienced” after. For some of the analyses they were assigned to a separate group labeled “became experienced before T5”. Blood was collected at the day of the first vaccine dose (T0), then after 21 days (the day of the second vaccine dose) (T1), and then at 14 days (T2), one month (T3), six months (T4), and 1 year (T5) following the vaccination. T5 also corresponds to the time point after the booster dose (IQR = 67-97 days). A total of 40 ml of blood was collected from each subject at each timepoint.

The protocol was approved by the ethics committee (full name: comité d’éthique hospitalo-facultaire universitaire de Liège) of Liège University Hospital (approval number 2021-54).

### Cell Mediated Immune Response to SARS-CoV-2 Infection

Cell mediated immune response was assessed by measuring the secretion of IFN-γ by peripheral blood lymphocytes using the QuantiFERON Human SARS-CoV-2 (Qiagen, Cat. No./ID: 626410). Briefly, blood was collected on four tubes: the control set including one positive and negative one negative tube and the two original Vacutainer tubes containing SARS- CoV-2 antigen 1 (Ag1) and SARS-CoV-2 antigen 2 (Ag2) formulated to activate CD4 T (by Ag1) and both CD4 T and CD8 T (by Ag2) lymphocytes in heparinized whole blood. After blood collection and mixing, tubes were incubated at 37°C for 16 to 24h. IFN-γ was measured in these plasma samples using CLIA on the DiaSorin LIAISON^®^ QuantiFERON^®^-TB Gold Plus (REF:311010) and was reported in International Units per ml (IU/ml). According to the data sheet provided by the manufacturer, early data suggested an INF-ɣ cutoff for positivity at 0.15 IU/mL.

### Assessment of Neutralizing Antibodies by Live-Virus Neutralization Assay

Neutralization assays were conducted in a specialized biosafety level 3 (BSL3) facility using a SARS-CoV-2 virus Wuhan-like variant (BetaCov/Belgium/Sart-Tilman/2020/1) isolated from a patient hospitalized in March 2020. Virus isolation, expansion, titration and SNT analysis were all performed using nonadherent sub-confluent Vero E6 cells (ATCC^®^ CRL-1586) grown in DMEM supplemented with 2% FBS and penicillin-streptomycin.

The virus stock was titrated in serial log dilutions to obtain a 50% tissue culture infective dose (TCID50) in 96-well culture plates. The plates were monitored daily using inverted optical microscope for five days to evaluate the presence of cytopathic effect (CPE) and the end-point titer was calculated according to the Reed & Muench method based on 2 x 3 replicates.

Serum test samples were heat-inactivated for 40 min at 56°C and two-fold serial dilutions, starting from 1:10 up to 1:320, were performed in triplicate in DMEM/FBS in 96-well culture plates. Sera dilutions (50 µl/well) were then mixed with an equal volume of pre-titrated viral solution containing 100 TCID50 of SARS-CoV-2 virus. The serum-virus mixture was incubated 1 h at 37°C in a humidified atmosphere with 5% CO2. After incubation, 100 μl of Vero cells’ suspension containing about 20,000 cells were added in each well (18). The plates were then re-incubated for 5 days. For each serum, the process was repeated twice by two independent trained people. After 5 days, CPE was evaluated under light microscopy by two independent persons. Serum dilutions showing CPE were considered as non-neutralizing (negative), while those showing no CPE were considered neutralizing/positive. Virus sero-neutralization titer was reported as the highest dilution of serum that neutralizes CPE in 50% of the wells (NT50). If results from the 2 duplicate plates were discordant, these samples were processed again in a subsequent SNT session. For all sera showing a NT50>1:320, a second process was made using higher dilutions (up to 1:20,480). Positive (NT50 = 1:160, from the Belgian National Reference Centre) and negative (saline) controls were inserted in each plate.

### Assessment of Total Anti-Spike IgGs

The DiaSorin LIAISON^®^ SARS-CoV-2 TrimericS IgG assay (DiaSorin, Stillwater, USA), a chemiluminescent immunoassay using magnetic particles coated with recombinant trimeric SARS-CoV-2 spike protein, was used for quantitative determination of IgG antibodies in human serum samples. The assay was performed on a LIAISON XL analyzer (DiaSorin) according to the manufacturer’s instructions. The IgG antibody concentration provided by the analyzer is expressed as Binding Antibody Units/ml (BAU/ml). The measurement range was 4.81 to 2080 BAU/ml. Values below 4.81 BAU/ml were reported as « < 4.81 BAU/ml ». Samples with high IgG antibodies (>2080 BAU/ml) were automatically diluted with the LIAISON^®^ TrimericS IgG Diluent Accessory. The manufacturer’s recommended dilution factor of 1:20 was used. The cut-off for positivity was ≥ 33.8 BAU/ml. Clinical sensitivity and specificity of this test were 98.7% and 99.5% respectively.

### Assessment of Total SARS-CoV-2 Anti-Nucleocapsid IgG Antibodies

Anti-SARS-CoV-2 nucleocapsid IgG antibodies were measured using electrochemiluminescent immunoassay (ECLIA) Elecsys^®^ anti-SARS-CoV-2 assay on Roche Cobas e801 (Roche Diagnostics, Basel, Switzerland) according to the manufacturer’s instructions. Results were expressed as a cutoff index (COI; signal sample/cutoff). The analyzer automatically calculates the cutoff based on the measurement of negative and positive calibrators. A cutoff index equal to or higher than 1.0 was interpreted as positive. Clinical sensitivity and specificity of this test were 99,5% and 99.8% respectively.

## Data Analysis

All the analyses were carried out in R version 4.1.1. All codes and data to reproduce the results are available at: https://github.com/tashkeev-alex/vaccination_study.

### Separating Individuals With Neutralizing Versus Non-Neutralizing Antibodies by Anti-Spike IgG

Logistic regression of neutralizing status at the last time point before the booster dose (i.e. T4, 6 months post-vaccine) on the log of anti-Spike IgG was used to determine the threshold immunoglobulin value that provides the best separation of the classes. Sensitivity and specificity of thе model were estimated by summarizing those across five stratified folds of cross-validation (no hyperparameter tuning or model selection was performed).

### Testing for Differences or Associations Among Immune and Clinical Parameters

In all corresponding procedures we used non-parametric statistics, i.e. either tie-corrected Spearman correlation coefficient or Kruskal-Wallis/Wilcoxon rank-sum tests. For the time points comparison we used unpaired testing. Indeed, the overlap between tested individuals is partial since not all individuals have data for all time points. Such a procedure does not invalidate the test but reduces its power. We used log-transformed anti-Spike IgG, NT50, and IFN-γ values. In multiple linear regression models, we centered and scaled the continuous predictors by two standard deviations allowing the comparison of coefficients for continuous and binary predictors on the same scale (19). In case of continuous dependent variables, we centered and scaled them in the same way. In case of multivariate dependent variables (anti-S IgG or NT50 across time points), we centered and scaled them across individuals within each time point.

## Results

### Cohort Characteristics, Demographics and Samples Collection

Our study included 208 consenting subjects among the CHU of Liège staff members who received the Pfizer–BioNTech BNT162b2 vaccine during February 2021. Characteristics of the cohort are summarized in **Table 1**. In total, 1145 samples were collected. Sampling was performed the day of first and second dose of the vaccine (T0 and T1), then 2 weeks, 4 weeks, 6 months and one year after the vaccination (T2, T3, T4, and T5) (**Table 2**; **Figure S1**). Between T4 and T5, participants got the 3rd boosting dose of the vaccine (IQR = 67-97 days before T5). 45/151 of naive participants donated blood at all time points while 69/151, 19/151, 16/151, and 2/151 donated blood 5, 4, 3, and 2 times respectively. In the experienced group, 13/57 of subjects donated blood at all time points while 27/57, 12/57, 4/57, and 1/57 donated 5, 4, 3 and 2 times respectively.

**Table 1:** Cohort description.

Characteristic	Experienced, N = 57 ^*1*^	naive, N = 151 ^*1*^
**# time points**		
2	1 (1.8%)	2 (1.3%)
3	4 (7.0%)	16 (11%)
4	12 (21%)	19 (13%)
5	27 (47%)	69 (46%)
6	13 (23%)	45 (30%)
**Sex**		
F	44 (77%)	120 (81%)
M	13 (23%)	28 (19%)
Unknown	0	3
**Age**	47 (37, 55)	42 (34, 53)
Unknown	0	3
**Smoking**	7 (12%)	23 (16%)
Unknown	0	3
**BMI**	24.1 (21.5, 27.6)	24.8 (21.2, 27.3)
Unknown	3	7
**Asthma**	4 (7.3%)	16 (11%)
Unknown	2	5
**Autoimmunity**	1 (1.8%)	8 (5.5%)
Unknown	2	6
**Immunodeficiency**	1 (1.9%)	2 (1.4%)
Unknown	3	6
**Blood Cancer**	0 (0%)	3 (2.1%)
Unknown	2	5
**Other Cancer**	2 (3.6%)	6 (4.1%)
Unknown	2	5

F, female; M, male; BMI, body mass index; IQR, interquartile range.

^1^n (%); Median (IQR).

**Table 2:** Timing of the sampling points.

Characteristic	T1, N = 208 ^*1*^	T2, N = 177 ^*1*^	T3, N = 183 ^*1*^	T4, N = 169 ^*1*^	T5, N = 55 *^1^*
# days after vaccination	21 (21, 21)	35 (35, 36)	49 (49, 52)	189 (189, 190)	350 (346, 352)
# days after previous time point	21 (21, 21)	14 (14, 14)	14 (14, 15)	140 (140, 140)	160 (157, 162)

Number of days after vaccination and after previous sampling, IQR, interquartile range.

^1^Median (IQR).

### Dynamics of Humoral Response to SARS-CoV-2 mRNA Vaccine BNT162b2

**Figure 1:**
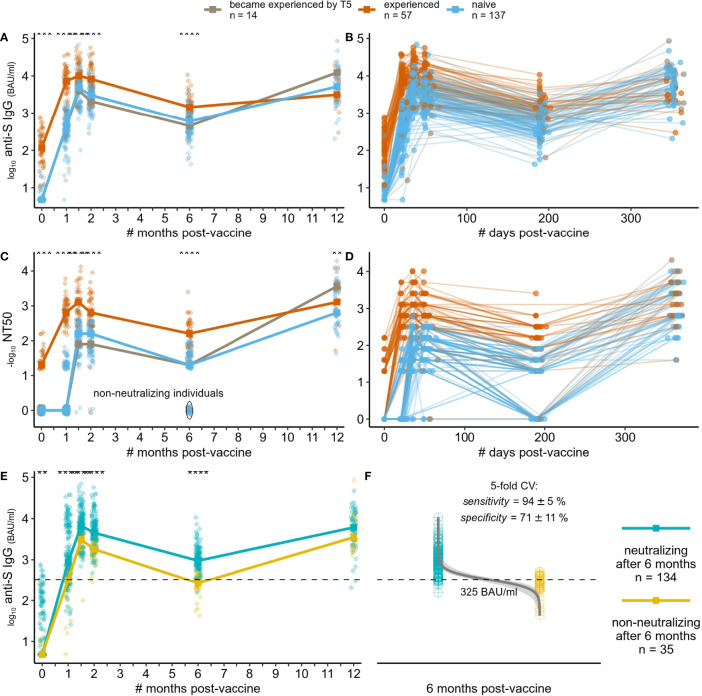
Dynamics of humoral immune response to SARS-CoV-2 mRNA vaccine BNT162b2. **(A, B)** dynamics of anti-Spike IgG (log10 BAU/ml) – median across individuals per approximate time point **(A)**, or for each individual **(B)** with exact time points; **(C, D)** dynamics of virus neutralization titer (-log10 values, zeroed if titer was less than 1/20) – median across individuals per approximate time point **(C)**, or for each individual with exact time points **(D)**; **(E)** same as in **(A)**, but grouping individuals by the ability to neutralize virus at the last time point [based on **(C)**]*;* **(F)** Logistic regression-derived boundary value of anti-Spike IgG level at the last time point separating individuals with no detectable neutralization at T4 from the others (inability to separate them by anti-Spike IgG at earlier time points is shown on **Figure S3**), mean ± standard deviation values of the model performance are shown. **: p <= 0.01 ,***: p <= 0.001, ****: p <= 0.0001.

We assessed the level of circulating trimeric Spike IgG in serum samples using DiaSorin LIAISON SARS-CoV-2 TrimericS IgG assay. The level of anti-Spike antibodies clearly discriminates between naive and experienced groups at T0 (day of the first vaccine dose), being 4.8 (IQR = 4.8-4.8) and 117 (IQR = 55.4-245) BAU/ml on median, respectively. The level of anti-Spike antibodies increased rapidly after the first vaccine dose in both groups but with higher titer in the experienced group over the naive one (497 BAU/ml in naive, 6630 BAU/ml in SARS-CoV-2 experienced). The anti-Spike circulating antibodies levels reached their maximum two weeks after the second vaccine dose and started declining two weeks later. This decrease continued over time between T3 and T4. The kinetics of anti-Spike antibodies level was similar between the naive and experienced groups, while the antibodies level itself was significantly higher in the experienced group over the naive group at all time points (**Figures 1A, B**, see **Figure S2** for anti-nucleocapsid IgG S/CO dynamics).

We measured neutralization capacities at all time points and all subjects using previously reported protocols (19). More than a half of SARS-CoV-2-experienced participants (35/57) showed a detectable neutralization (mostly 1:20) at the day of the vaccine first dose administration (T0), in contrast to subjects with no prior SARS-CoV-2 infection (naive group) (**Figure 1C**, **D**). At T1, median neutralization titer increased substantially to 1:640 in the experienced group, while no increase was observed in the vast majority (139/151) of the naive group. Two weeks after the second vaccine dose (T2), almost everyone developed a neutralizing ability, regardless of a previous exposure to the virus. Though, the neutralization capacity at T4 (6 months after vaccination) decreased to a non-detectable level in 35 naive individuals, namely in those who also showed poor response to the first vaccine dose. Importantly, after the booster administration, everyone has acquired the neutralization capacity again (6/35 also became experienced before T5, 13/35 were still naive, while others were not measured).

In clinical practice, measuring antibodies neutralization capacities is complicated and time consuming. Therefore, and since measuring anti-Spike IgG level is routinely used, we modeled the ability to use anti-Spike antibodies levels to predict whether a person still has some neutralization capacity at some reasonably far time point after vaccination (6 months in the case of our study) (**Figures 1E, F**). Logistic regression classifier allowed the separation of individuals with no detectable neutralization at T4 and from the others by the anti-Spike IgG at T4 (sensitivity: mean=94%, std=5%; specificity: mean=71%, std=11% across five stratified folds of cross-validation). The decision value estimated on the whole dataset was 325 BAU/ml. Using anti-Spike IgG level at earlier time points did not allow such classification (**Figure S3)**.

### Association of BNT162b2-Induced Anti-SARS-CoV-2 Neutralizing Antibodies With Participants’ Clinical Parameters

**Figure 2:**
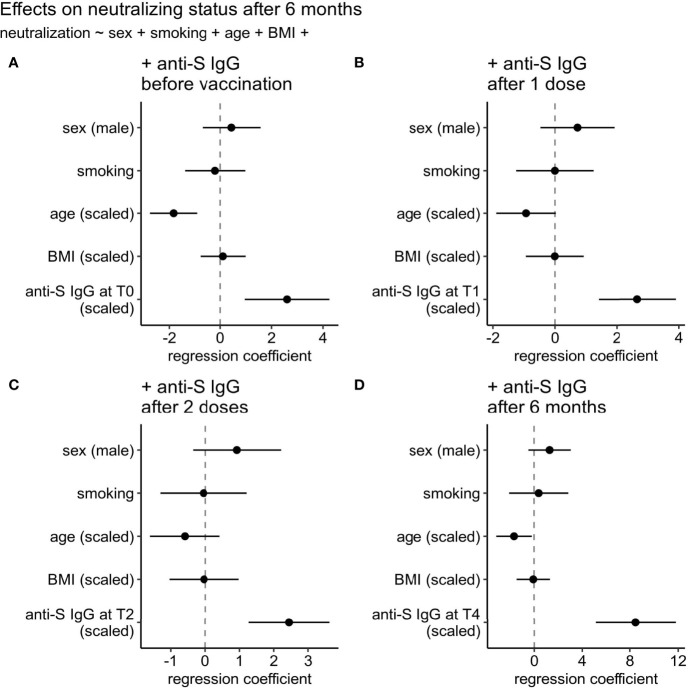
Effects of clinical parameters on neutralizing status at six months after 1^st^ vaccination dose. **(A)** anti-Spike IgG level at 1^st^ time point (T0: day of the 1^st^ vaccine dose), **(B)** anti-Spike IgG level at T1 time point (after 1^st^ vaccine dose), **(C)** anti-Spike IgG level at T2 time point (after 2^nd^ vaccine dose), or **(D)** anti-Spike IgG level at T4 (6 months after vaccination). Regression coefficients for all predictors can be compared on the same scale since continuous predictors were centered and scaled by two standard deviations.

Because of the variable level of the anti-Spike circulating antibodies’ proxies of quantity and quality (represented by binding antibody and live-virus neutralization assays) between vaccinated (naive and experienced) subject, we assessed the association of total antibodies as well as neutralizing status of individuals at the last time point before the boost (T4) with a reasonable set of available clinical parameters such as sex, smoking status, age, and body mass index (BMI) in a single model containing also one of the parameters representing the anti-Spike IgG level (**Figure 2**). By the latter we considered anti-Spike IgG level before vaccination (T0) (**Figure 2A**), after 1 dose vaccination (T1) (**Figure 2B**), after 2 doses vaccination (T2) (**Figure 2C**), or 6 months after vaccination (T4) (**Figure 2D**). Among all tested clinical parameters, only age was associated with the neutralizing status in three of the four tested models (decreasing chances to have neutralizing capacity), and even its effect size was far less (1.4-5.1x) than that of the parameter representing anti-Spike IgG level. No significant effect of sex, smoking status, and BMI were detected.

### Cellular Immune Response to SARS-CoV-2 mRNA Vaccine BNT162b2

**Figure 3:**
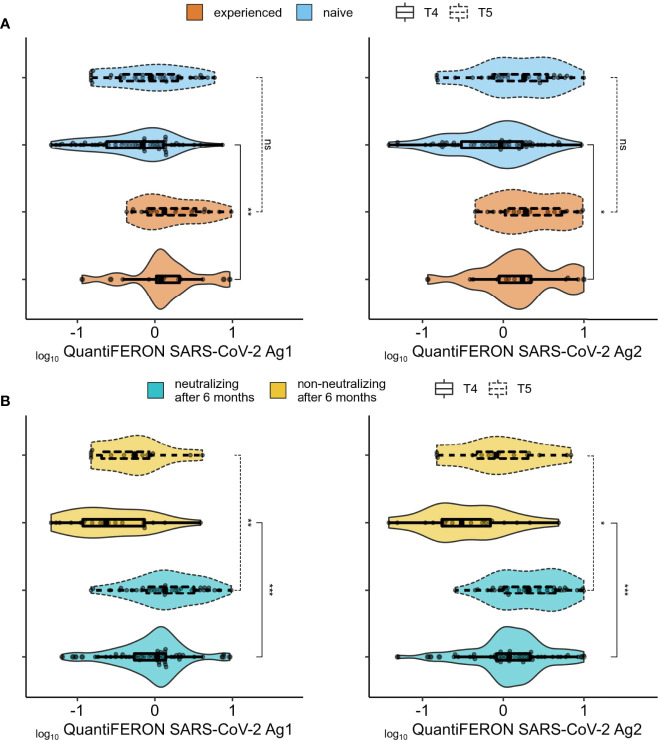
Association between cellular and humoral responses. **(A)** Comparison of cellular immune response (QuantiFERON-SARS-CoV-2 Ag1 (left panel) and QuantiFERON-SARS-CoV-2 Ag2 (right panel) between SARS-CoV-2 experienced (orange) and naïve (blue) groups. **(B)** Same as **(A)** but comparing individuals with no detectable neutralization at the 6 months after 1^st^ vaccine dose (yellow) and those with neutralizing titers (aquamarine) at the same time point. ns (not significant): p > 0.05, *: p <= 0.05, **: p <= 0.01, .***: p <= 0.001.

**Figure 4:**
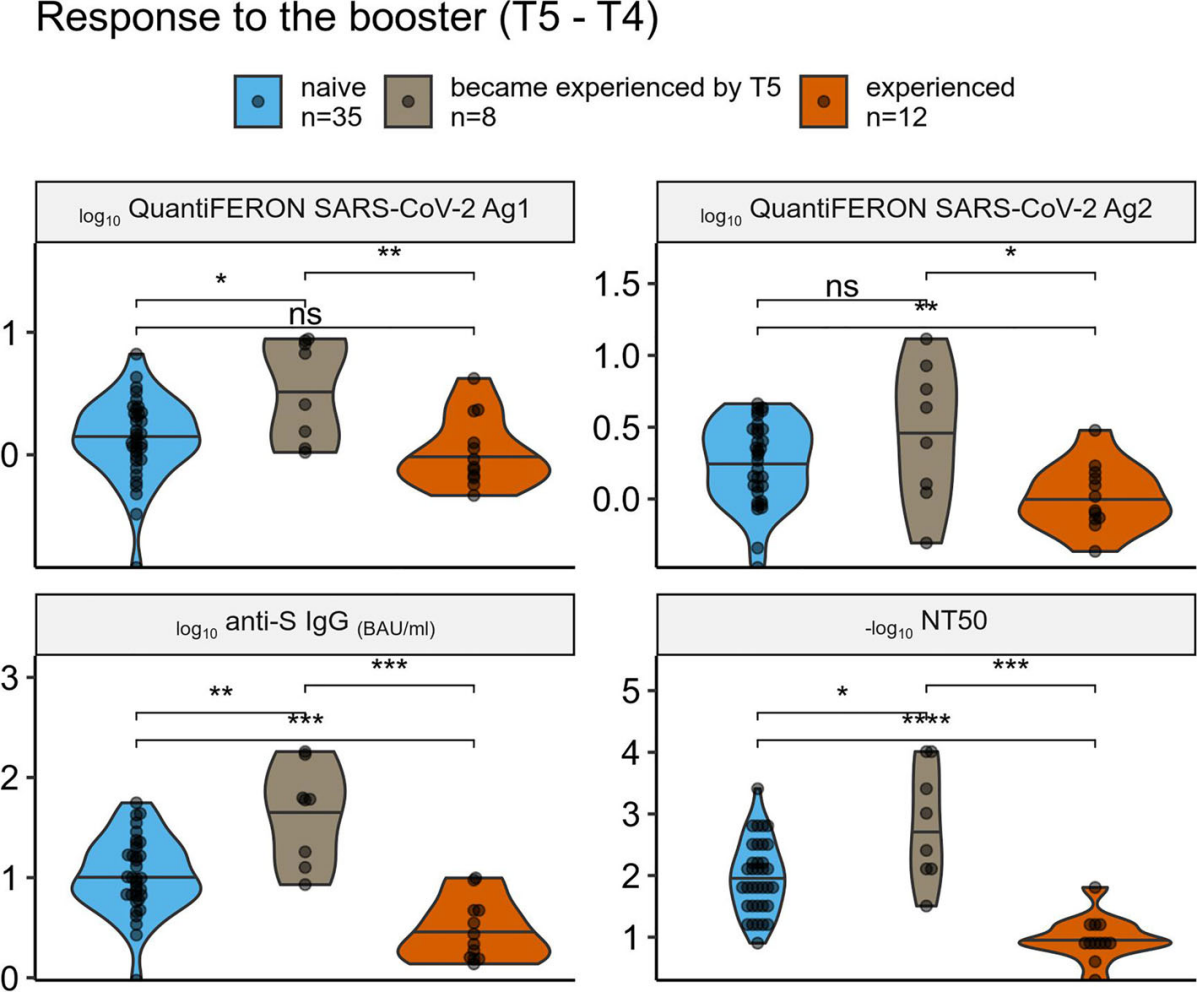
Comparison of the vaccine response among individuals grouped by their prior SARS-CoV-2 exposure. Comparison of cellular (top panels) and humoral (bottom panels) immune response to the 3rd (boosting) vaccine dose across SARS-CoV-2 naive, recently experienced, and experienced-long-time-ago groups. ns (not significant): p > 0.05, *: p <= 0.05, **: p <= 0.01, ***: p <= 0.001, ****: p <= 0.0001.

To evaluate T-cell specific immune response to BNT162b2 vaccine, heparinized whole blood was stimulated with SARS-CoV-2 specific peptides contained in the QuantiFERON-SARS-CoV-2 tubes designed to activate both CD4T and CD8T cells. IFN-γ was then measured by CLIA. Since this assay was only commercially available after we completed the collection of samples at T0 to T3, we only assayed cellular immune response at T4 and T5 (**Figures 3**, **4**) with 62 naive plus 17 experienced individuals for the former, and 35 naive and 20 experienced for the latter (8/20 became experienced between T4 and T5, while nobody became experienced between T0 and T4). At T4, experienced individuals had higher cellular immunity compared to naive (**Figure 3A**), while this difference disappears after the boost at T5. For both T4 and T5, the between-groups difference in IFN-γ secretion was larger and more significant when subjects were grouped by their neutralization capacity at 6-months after the first dose (T4) (**Figure 3B**), then by grouping according to prior virus exposure. We next investigated if there was any association between IFN-γ secretion, anti-Spike total antibodies and neutralizing antibodies titers. Correlation between anti-S IgG and IFN-γ at T4 was more pronounced in the experienced group for both Ag1 and Ag2 (mean rho = 0.53 in experienced vs 0.33 in naive), while the correlation of IFN-γ and neutralizing antibodies titer was similar between naive and experienced (mean rho = 0.53 in experienced vs 0.49 in naive) (**Figure S4**). Interestingly, at T5 (after the booster and 1-year after the first dose) there was no correlation between cellular and humoral responses for the experienced group, while for naive subjects, results were quite similar to those at T4.

### Immune Response to the SARS-CoV-2 mRNA Vaccine Boosting Third Dose

We had 55 individuals with all the data for T5 and T4, and thus we were able to analyze the difference between the response to the third boosting vaccine dose among individuals’ subgroups – naive, those who became experienced between T4 and T5, and those who was experienced already before the first dose (**Figure 4**). Despite the small sample size of each group (n = 35, 8, and 12, respectively), there was a significant difference in terms of humoral response, with experienced individuals having the lowest response that could be explained by stronger immunity at T4, and the “recently experienced” individuals having the highest response that could reflect their recent “natural” boosting of the immunity by the SARS-CoV-2 infection. Difference in terms of cellular response was less pronounced, however there was a tendency similar to the one observed for the humoral response.

## Discussion

Our study confirms previous evidence for an earlier, stronger and more persistent humoral immune response in individuals previously infected with SARS-CoV-2 versus naïve individuals following vaccination with the BNT162b2 mRNA vaccine (20). The anti-spike antibodies and neutralization capacity levels six months after vaccination protocol were significantly higher in SARS-CoV-2 experienced HCWs compared to naïve HCWs. We also observed a higher cellular immune response six months following vaccination in SARS-CoV-2 experienced HCWs, although the difference was less remarkable. Reassuringly, most participants, including SARS-CoV-2 naive individuals, had a detectable cellular immune response to SARS-CoV-2 six months after vaccination. These findings are in line with a recent observation from Samanovic and colleagues who also reported more pronounced differences in humoral over cellular responses between individuals SARS-CoV-2-naive versus recently SARS-CoV-2 experienced subjects (21).

Besides the impact of SARS-CoV-2 infection history on immune response to BNT162b2 mRNA vaccine, we observed a significant association between age and persistence of humoral response. The more elderly is a participant, the less durable was the humoral response. This is in line with results from other studies demonstrating a similar decrease of anti-SARS-CoV-2 antibodies in all age groups a few months after the second vaccine dose, especially among 65 years-old or older persons (22). Regarding cellular immune response, although not statistically significant, we observed a trend towards a negative impact of age.

Our results confirm previous findings indicating that anti-spike antibodies are very well correlated with neutralizing antibodies (22, 23). This may allow to set a threshold of anti-spike antibodies predicting neutralization capacity with a high sensitivity. Although correlates of protection from SARS-CoV-2 are not fully defined yet, Khoury and colleagues showed that neutralization level is highly predictive of immune protection, reinforcing the results of other reports suggesting that neutralization titer is an important predictor of vaccine efficacy (24–26). Our findings may thus have implications to better identify individuals with strong protective immunity or individuals with lower predicted immune protection who could be at higher risk of disease progression based on anti-spike antibodies level. For instance, it could contribute to better define patients who are at high risk of developing severe COVID-19 and who could benefit the most from anti-SARS-CoV-2 mAb products or other therapeutic options that are now available (27, 28). Available stocks of mAbs that retained activity against Omicron are extremely limited in most settings, while many vulnerable patients can be considered as eligible for this treatment. Predictors of remaining neutralization capacity may help clinicians with the difficult selection of patients for whom this intervention would be most beneficial. Nevertheless, additional studies are needed to better determine the usefulness of anti-spike antibodies for COVID-19 treatment prioritization.

Regarding booster dose, we showed that it induced a significant increase of both the humoral and cellular immune responses. Importantly, the benefit of the booster dose was lower in experienced individuals who had higher persisting levels of humoral or cellular immunity before the booster dose. In addition, individuals who were infected during the course of the study got higher responses of cellular and humoral immunity after the booster doses. This is not surprising as those responses reflect both the effect of the booster dose and the effect of the breakthrough infection.

These two observations underline a strong impact of SARS-CoV-2 infection on the magnitude and the persistence of vaccine responses and suggest that naive individuals should be prioritized for additional booster doses.

The humoral immune response as measured through quantification of anti-spike IgG or neutralizing antibodies correlated with cellular immune responses. Interestingly, this correlation is preserved also after the boosting dose only in the group of participants without a history of SARS-CoV-2 infection, indicating that the level of anti-spike antibodies in this group may not only predict the level of neutralizing antibodies but also the level of cellular immunity as well.

In conclusion, our data strongly reinforce the relevance of previous SARS-CoV-2 infection for understanding vaccine immune responses. It may have implications for personalizing mRNA vaccination regimens used to prevent severe COVID-19 and reduce the impact of the pandemic on the healthcare system. More specifically, it may help prioritizing vaccination, including for the deployment of additional booster doses. Naive individuals benefit the most from the booster dose.

## Data Availability Statement

The original contributions presented in the study are included in the article/**Supplementary Material**. Further inquiries can be directed to the corresponding authors.

## Ethics Statement

The studies involving human participants were reviewed and approved by comité d’éthique hospitalo-facultaire universitaire de Liège, approval number 2021-54. The patients/participants provided their written informed consent to participate in this study.

## Author Contributions

Conceptualization: DD, SR, GD; methodology: DD, SR, GD; formal analysis: SD, AT, DD, SR, GD; investigation: SD, AT, ME, NM, HP, YT, LL, CFL, BP, CL, MW, MMn, NF, LL, M-PH; writing-original draft preparation, SD, AT, DD, SR, GD; review and editing: all authors; supervision: LG, FB, SR, M-PH, MMo, CM, GD, DD; funding acquisition: GD, SR, MMo. All authors have read and agreed to the published version of the manuscript.

## Funding

Léon Fredericq Foundation (To GD and MM), FNRS (Fonds National de la Recherche Scientifique) [To SR, grant N° PER/PGY H.P030.20)]. AT is aspirant FNRS (PhD fellow), GD is an FNRS postdoctoral clinical master specialist and SR is an FNRS Senior Research Associate. The funders had no role in study design, data collection and analysis, decision to publish, or preparation of the manuscript.

## Conflict of Interest

Authors FB and LG are the inventors of the device used in the saliva collection kit. This device was patented (EP20186086.3) and produced by Diagenode (Seraing, Belgium) under a commercial agreement with the University of Liège. This does not alter the adherence to all journal policies on sharing data and materials.

The remaining authors declare that the research was conducted in the absence of any commercial or financial relationships that could be construed as a potential conflict of interest.

## Publisher’s Note

All claims expressed in this article are solely those of the authors and do not necessarily represent those of their affiliated organizations, or those of the publisher, the editors and the reviewers. Any product that may be evaluated in this article, or claim that may be made by its manufacturer, is not guaranteed or endorsed by the publisher.
