# Supplementary material for: Kinetics and Persistence of the Cellular and Humoral Immune Responses to BNT162b2 mRNA Vaccine in SARS-CoV-2-Naive and -Experienced Subjects: Impact of Booster Dose and Breakthrough Infections
Source: Front Immunol. 2022 May 31;13:863554. doi: 10.3389/fimmu.2022.863554 (PMC9195074; doi:10.3389/fimmu.2022.863554)
Supplement: Supplementary file 1 [file DataSheet_1.pdf]

Supplementary material

Kinetics and persistence of the cellular and humoral immune responses to BNT162b2 mRNA vaccine in SARS-CoV-2-naive and -experienced subjects: impact of booster dose and breakthrough infections

Salomé Desmecht[1,2¶], Aleksandr Tashkeev[1¶], Majdouline El moussaoui[2,3], Nicole Marechal[3], Hélène Perée[1], Yumie Tokunaga[1], Celine Fombellida-Lopez[2], Barbara Polese[4], Céline Legrand[4], Marie Wéry[1], Myriam Mni[1], Nicolas Fouillien[1], Françoise Toussaint[7], Laurent Gillet[5], Fabrice Bureau[4], Laurence Lutteri[6], Marie-Pierre Hayette[7], Michel Moutschen[2,3], Christelle Meuris[3], Pieter Vermeersch[8], Daniel Desmecht[9&], Souad Rahmouni[1&], Gilles Darcis[2,3&]

Supplementary figures and legend

Figure S1

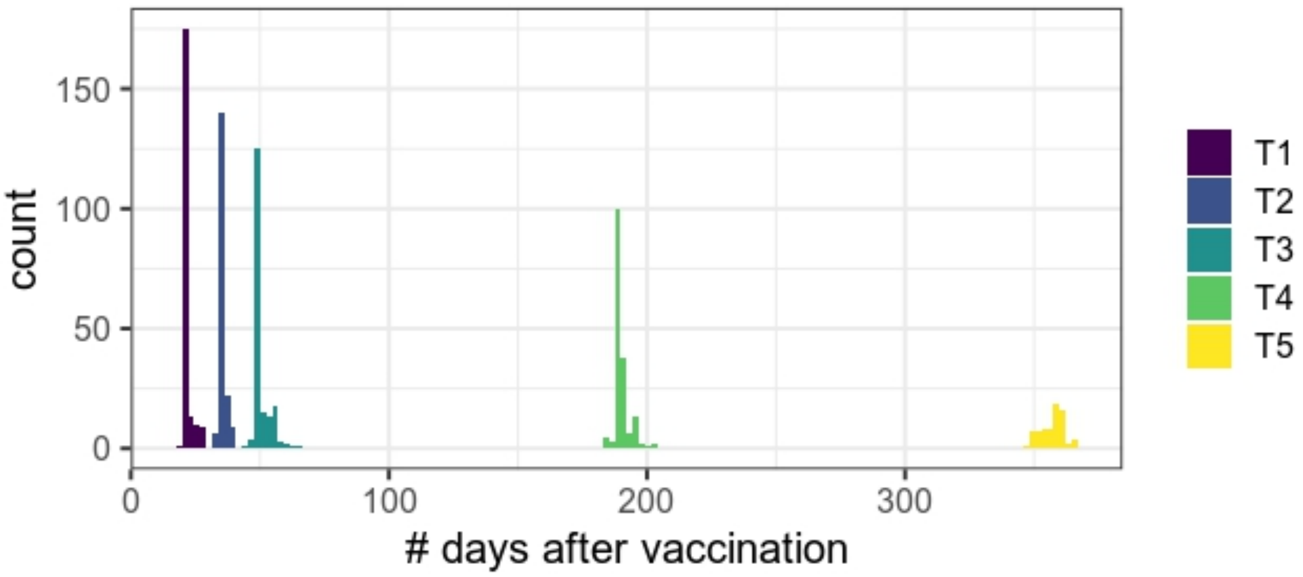

Figure S1. Histogram of number of days after the 1st dose across time points.

Figure S2

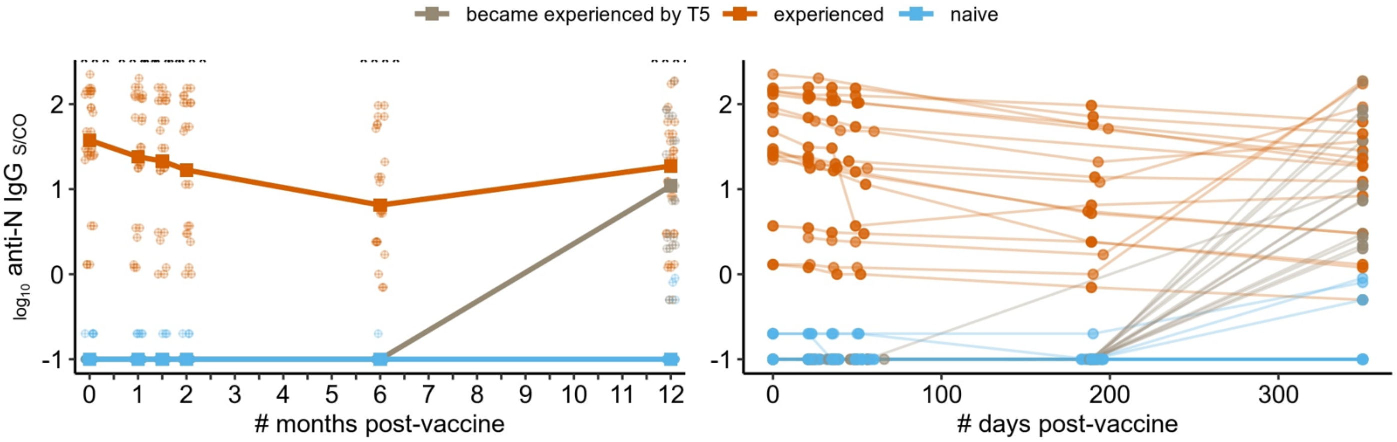

Figure S2. Dynamics of anti-Nucleocapsid IgG (log10 S/CO, signal-to-cutoff ratio) **Left -** median across individuals per approximate time point, **Right** - for each individual with exact time points.

Figure S3

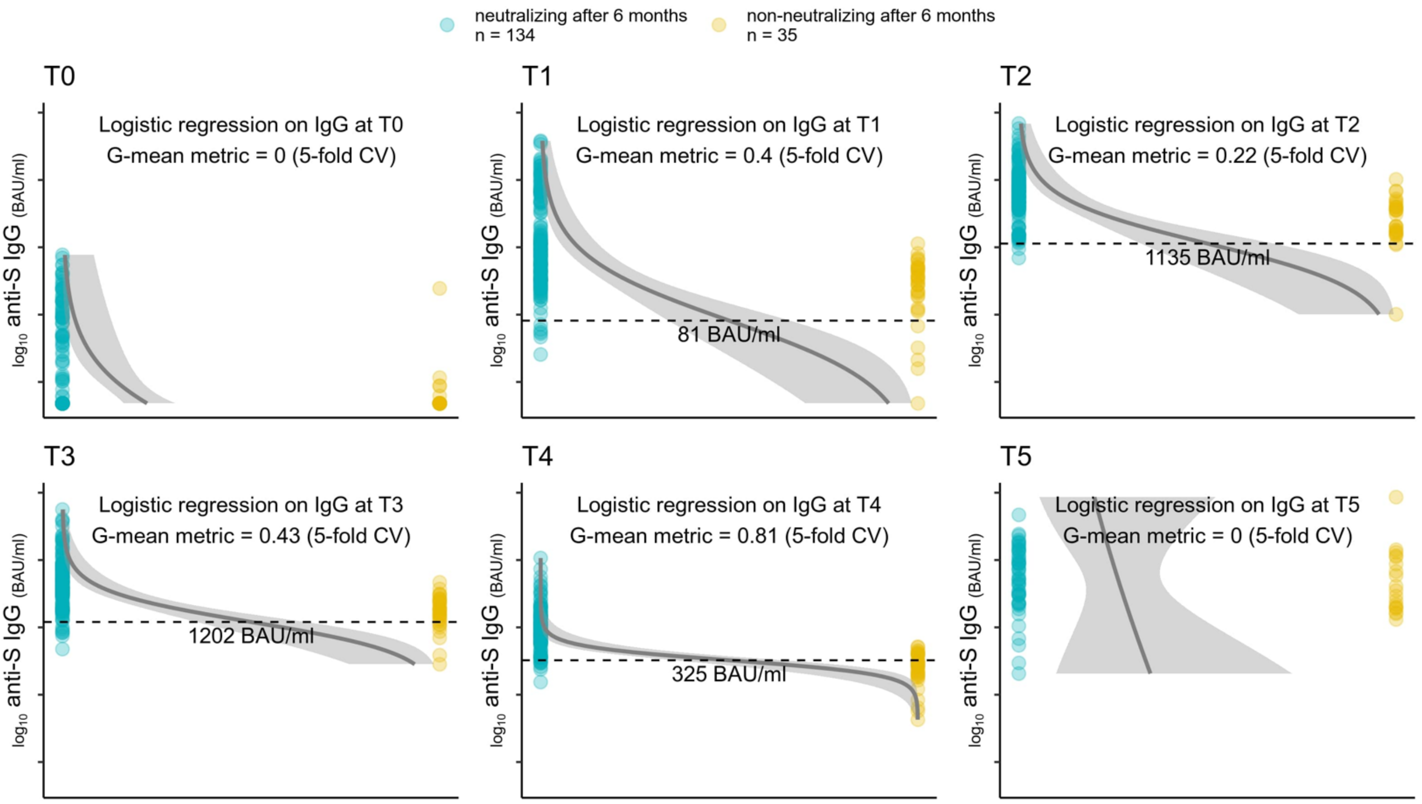

Figure S3. Separation of individuals with no detectable neutralization at T4 from the others by anti-Spike IgG across timepoints. G-mean metric = geometric mean of sensitivity and specificity. CV = cross-validation.

Figure S4

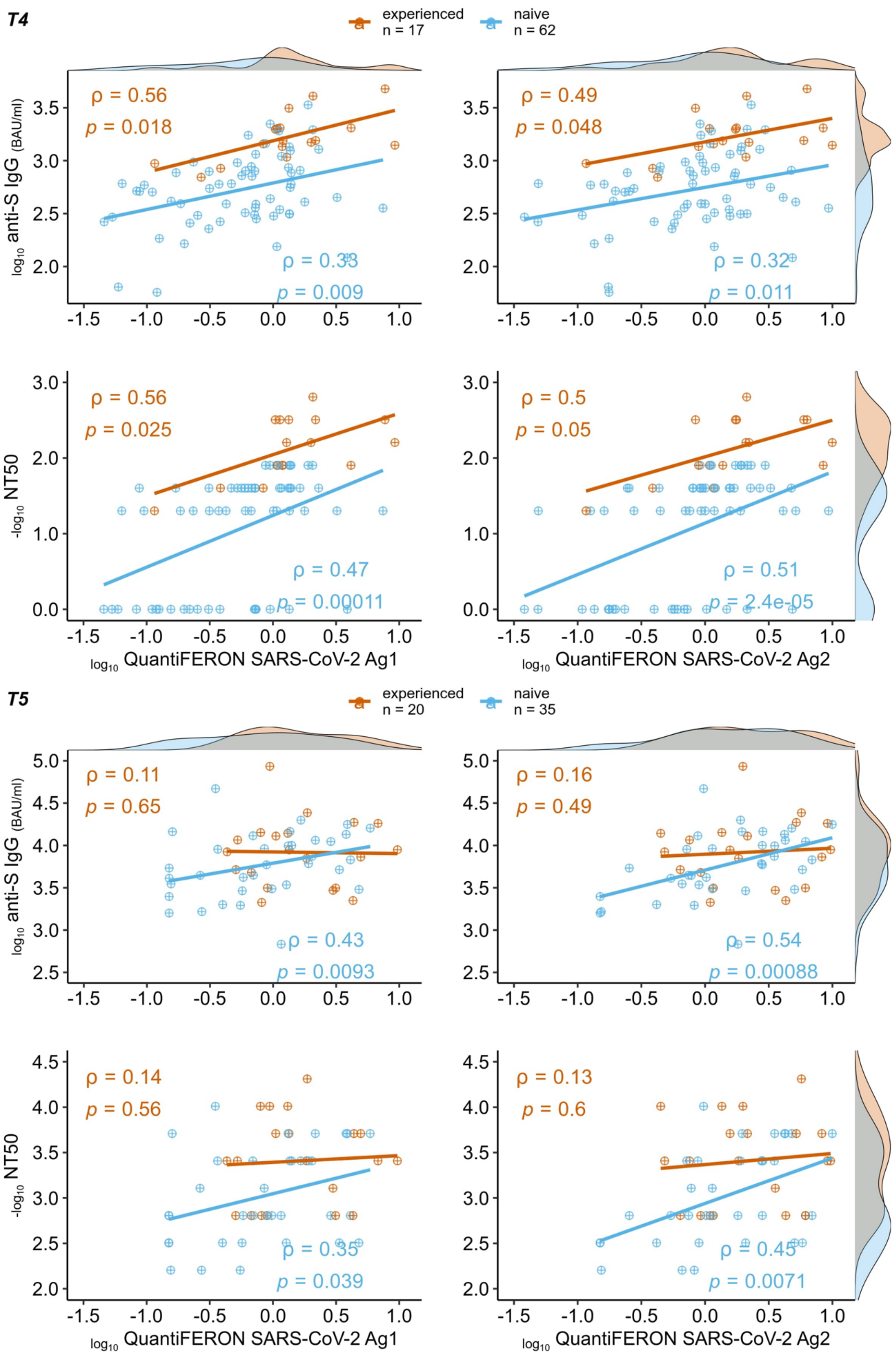

T4
experienced n = 17
naive n = 62
ρ = 0.56
p = 0.018
ρ = 0.33
p = 0.009
ρ = 0.49
p = 0.048
ρ = 0.32
p = 0.011
log10 anti-S IgG (BAU/ml)
ρ = 0.56
p = 0.025
ρ = 0.47
p = 0.00011
ρ = 0.5
p = 0.05
ρ = 0.51
p = 2.4e-05
-log10 NT50
log10 QuantiFERON SARS-CoV-2 Ag1
log10 QuantiFERON SARS-CoV-2 Ag2
T5
experienced n = 20
naive n = 35
ρ = 0.11
p = 0.65
ρ = 0.43
p = 0.0093
ρ = 0.16
p = 0.49
ρ = 0.54
p = 0.00088
log10 anti-S IgG (BAU/ml)
ρ = 0.14
p = 0.56
ρ = 0.35
p = 0.039
ρ = 0.13
p = 0.6
ρ = 0.45
p = 0.0071
-log10 NT50
log10 QuantiFERON SARS-CoV-2 Ag1
log10 QuantiFERON SARS-CoV-2 Ag2

Figure S4. Correlation between humoral and cellular responses at T4 and T5. Spearman correlation (rho) between anti-Spike IgG level (top panels) or neutralization titer (bottom panels) with cellular response represented by QuantiFERON-SARS-CoV-2 Ag1 (left) or Ag2 (right) at T4 and T5.
